# *IL-4, IL-17* and *CD163* Immunoexpression and* IL-6* Gene Polymorphism in Chronic Hepatitis C Patients and Associated Hepatocellular Carcinoma

**DOI:** 10.31557/APJCP.2021.22.4.1105

**Published:** 2021-04

**Authors:** Tarek Aboushousha, Marine Emad, Gina Rizk, Khaled Ragab, Olfat Hammam, Rabab Fouad, Noha Said Helal

**Affiliations:** 1 *Department oF Pathology, Theodor Bilharz Research Institute, Giza, Egypt. *; 2 *Faculty of Biotechnology, October University for Modern Sciences and Arts, Giza, Egypt. *; 3 *Department of Hepatology and Gastroenterology, Theodor Bilharz Research Institute, Giza, Egypt. *; 4 *Department of Hematology, Theodor Bilharz Research Institute, Giza, Egypt. *

**Keywords:** IL-4, IL-17, CD136, IL-6, HCV, HCC

## Abstract

**Objective:**

To assess the expression of *IL-4, IL-17* and* CD-163* as well as study of *IL6-572 C/G* gene polymorphism in chronic HCV and HCC on top of HCV.

**Methods:**

Sixty HCC specimens and 60 adjacent hepatic tissue with HCV of different grades of necro-inflammation and different stages of fibrosis. In addition to 55 HCV, 60 HCC and 50 healthy venous blood samples for evaluation of *IL6-572 C/G *gene polymorphism.

**Results:**

high expression of *IL-4, IL-17 *and *CD163* in higher grades of activity, late stages of fibrosis and higher degrees of steatosis of *HCV. IL-4* and *CD163 *showed higher expression in advanced grades of HCC, while *IL-17* more expressed in lower grades. No significant difference in *IL6-572 C/G* gene polymorphism among studied groups regarding G/C, G/G, C/C frequencies or G and C allele’s frequencies.

**Conclusion:**

*IL-4, IL-17* and *CD163* were associated with HCV severity. Their expression in HCC suggests their important role in HCC development. Blocking of these proteins may be a good target to control inflammation in HCV and can hinder progression to cirrhosis then to HCC. On the other hand, *IL6-572* promoter gene polymorphism is neither associated with HCV infection nor with HCC development and its progression.

## Introduction

Liver cancer is predicted to be the sixth most commonly diagnosed cancer and the fourth leading cause of cancer death worldwide in 2018, with about 841,000 new cases and 782,000 deaths annually (Bray et al., 2018). Hepatocellular carcinoma (HCC) comprises 75%-85% of primary liver cancer cases. Most cases of HCC occur on a background of chronic inflammation mainly due to exposure to hepatitis viral infection and cirrhosis (Farazi and DePinho, 2006). Hepatitis C virus (HCV) was a major cause of liver disease worldwide, leading to progressive fibrosis, potential development of cirrhosis and hepatocellular carcinoma (HCC) (Liang et al., 2000).

In Egypt, Liver cancer is the first most common cancer and the first leading cause of cancer-related deaths; with chronic HCV infection is likely the predominant cause for the development of HCC (Bray et al., 2018). 

Cytokines are produced by a broad range of cells, including immune cells like macrophages, B lymphocytes, T lymphocytes and mast cells, as well as endothelial cells, fibroblasts and various stromal cells (DePace and Colombo, 2019). In the liver; cytokines co-ordinate physiologic processes such as liver growth and regeneration, as well as, pathologic processes as inflammation during viral liver disease, liver fibrosis, and cirrhosis (Hammad et al., 2013). Cytokines can recognize HCV-infected hepatocytes and can regulate the immunological and inflammatory responses, and viral clearance, but they have also been implicated in the hepatocellular injury seen in the majority of chronically infected patients (Mourtzikou et al., 2014), which may be the first step in inflammation-based carcinogenesis. In addition, cytokines may trigger the hepatocarcinogenesis itself through growth signaling, angiogenesis and invasive metastasis (Capone et al., 2010). 

Interleukin-4 (IL-4) is produced by T helper (Th)-2 cells and has a major role as a mediator and modulator of immune and inflammatory responses (Lu et al., 2014) and regulates cell proliferation, apoptosis, and expression of numerous genes in lymphocytes, macrophages, and fibroblasts (Luzina et al., 2012).

Interleukin-17 (IL-17) is produced by T helper (Th)-17 cells and has been reported to trigger tissue inflammation and damage in various chronic diseases (Liu et al., 2016), including hepatic inflammation and liver cirrhosis (Rong et al., 2009), inflammatory bowel disease, rheumatoid arthritis, and multiple sclerosis (Axtell et al., 2010). IL-17 also is related to many human cancers, including gastric cancer, breast cancer, and ovarian cancer (Yang et al., 2016). 

Cluster of differentiation (CD) 163 is one of the M2 lineage (alternatively activated macrophages). It is a lineage-specific hemoglobin-haptoglobin scavenger receptor expressed exclusively on monocytes and macrophages and up-regulated in conditions with macrophage activation (Schaer et al., 2005). It is shed from the macrophage surface into the circulation and found in the blood as soluble CD163 (sCD163) (Kowal et al., 2011).

IL-6 is a multifunctional cytokine that is recently known to be an important constituent of cancer-associated cytokine complex which ultimately results in a systemic immune stimulation together with cancer-induced immune suppression that eventually protects the cancer cells (Lippitz and Harris, 2016). Although the relation between IL6 and HCC development is still unclear, yet several studies have revealed that HCC progression is solely dependent on the extend of liver inflammation, hence the balance between pro-inflammatory and anti-inflammatory cytokines is the key ingredient for controlling the disease progression; this means that *IL6* gene polymorphism could result in disturbance in this balance and development of the disease (Sghaier et al., 2017).

Assessment of immune cytokines is necessary to better understand the role of immune-inflammatory responses that determine patient outcomes.

This study aims to estimate immunohistochemical expression of *IL-4, IL-17*, and* CD-163* as well as study of *IL6-572 C/G* gene polymorphism in chronic HCV and HCC on top of HCV to determine the possibility of their use as therapeutic targets for chronic HCV to control progression to cirrhosis then to HCC.

## Materials and Methods

*Specimens*

For immunohistochemical staining technique; archival liver blocks from 60 cases of HCC associated with HCV were included in this study. For each case; two sections were cut, one from the tumor and the other from non-tumor area. For Genomic DNA extraction and genotyping; peripheral blood samples were obtained from 55 HCV patients, 60 HCC patients, and 50 healthy persons. HCV positivity was confirmed by Serological tests for HCV antibodies and PCR of HCV-RNA.

All procedures were done at the Pathology department and Central lab. Department, Theodor Bilharz Research Institute, Giza, Egypt.

*Tissue sectioning*

Paraffin sections from different hepatic lesions were cut in a thickness of 3-5 µm and spread on positively charged glass slides for better adherence. Sections were stained using hematoxylin and eosin stain (H&E stain) for routine examination grading and staging of hepatitis activity. Sections were also stained by Masson’s trichrome stain for assessment of fibrosis stage (according to METAVIR score). 

*Immunohistochemical staining*

Formalin-fixed paraffin sections were processed for immunohistochemical (IHC) analysis of IL-4, IL-17, and CD163 as follows: IHC examinations were carried out on 3 mm thick sections. Antigen retrieval was performed with 10 ml sodium citrate buffer, pH 6.0, at 90°C for 30 min. Sections were incubated in 0.03% hydrogen peroxide for 10 min at room temperature, to remove endogenous peroxidase activity, and then in blocking serum (0.04% bovine serum albumin, A2153, Sigma-Aldrich, Shanghai, China, and 0.5% normal goat serum X0907, Dako Corporation, Carpinteria, CA, USA, in PBS) for 30 min at room temperature. Anti-IL-4, Anti-IL-17 and Anti CD163 antibodies were used at a dilution of 1:200. The antibody was incubated overnight at 4°C. IL-4 mouse monoclonal antibody: Sc-12723 kit (Santa Cruz, USA), IL-17 rabbit polyclonal antibody: RC0017 kit (Medialysis, USA), and CD163 (GHI-61) mouse monoclonal antibody: (Santa Cruz, USA). Sections were then washed three times for 5 min in PBS. Non-specific staining was blocked with 0.5% casein and 5% normal serum for 30 min at room temperature. Finally, staining was developed with diaminobenzidine substrate and sections were counterstained with hematoxylin.

*Interpretation of Immunostaining*

The sections were examined by using light microscope (Scope A1, Axio, Zeiss, Germany). Photomicrographs were taken using a microscope-camera (AxioCam, MRc5, Zeiss, Germany).

Semi-quantitative analysis of *IL-4, IL-17*, and *CD163* expression was then estimated in referring to the intensity of cytoplasmic immunostatining scoring from zero to 3 as (0: no staining, 1: weak, 2: moderate, and 3: strong). Thus, cases with moderate and strong immunostaining were considered positive. Also, the percentages of cells were counted with positive expression in 5 successive high power fields. 

A total score from 0 to 300 was calculated by multiplying the score of intensity by the percentage of positive cells. Cases with a total score equal to or less than 10 are considered negative; cases with a total score from 11 to 100 were considered of low expression. Cases with a total score of 151 to 300 were considered of high expression. 

*Genomic DNA extraction*

Genomic DNA was obtained using the QIAamp DNA Mini Kit (Qiagen; catalog No.: 51104). 5ml peripheral venous whole blood was collected in a sterile vacuum tube containing EDTA for genomic DNA extraction by means of standard protocol using proteinase K. Lysis of red blood cells was done 3 times using lysis buffer. Afterwards, Sodium dodecyl sulfate (SDS) 10% and 10 ul proteinase K in the presence of guanidine HCL were added to treat the remaining white cells for a short incubation time (10 minutes at 56^o^C) in order to inactivate all nucleases. Cellular nucleic acids then bind to a special glass fibers pre-packed in high pure purification filter tube and a series of “wash and spin” steps were executed using 500 ul Buffer AW1 and 500 ul Buffer AW2 for getting rid of PCR impurities. Finally, elution buffer (200 ul Buffer AE) was added and incubation was done for 1 min at 15-25^o^C to release the nucleic acid from the glass fiber.

*IL-6 genotyping*

*IL-6 C/G* gene polymorphism (rs 1800796) was detected using Taq Man SNP genotyping assay. This assay consists of a single, ready to use tube that contain two sequence –specific primers for amplifying the polymorphism of interest together with two allele-specific Taq Man minor groove binder (MGB) probes for detecting the alleles for the specific polymorphism of interest. Each probe has a reporter dye; VIC dye is linked to the 5’ end of allele C probe while FAM dye is linked to the 5’ end of allele G probe. Each PCR reaction contained 2.5 ul of diluted DNA (5 ng/ul), 12.5 ul of 2× TaqMan Universal PCR Master Mix, 1.25 ul of 20× TaqMan SNP Genotyping Assay Mix and 8.75ul of Distilled water (DW). This PCR reaction was carried out in a thermal cycler using ABI 7500. Finally, a threshold is set at 0.1 for analysis; using ABI Prism “genetic analyzer”. This comprises quantitation of the amplified PCR product (DNA fragments) as well as determining the size of the fragments by comparing them to fragments contained in a size standard.

*Statistical analysis*

The statistical analysis was done using (SPSS software program, version 20). Comparing the results was done with analysis of variance (ANOVA) and student t-test to compare IL-4, IL-6 and IL-17 scores in different groups. Results were given as mean ±SD with 95% confidence interval. In addition to using the alleles and genotype frequencies and percentage for Categorical and non-parametric variables for *IL-6* gene polymorphism. Distribution of negative and positive cases was studied with cross tables (Ficher’s exact Chi square-test). To investigate a possible correlation of IL-4, IL-6 and IL-17 scores with tumor grade and stage, the Spearman rank correlation coefficient was used. In all tests, P <0 .05 was considered to indicate significant.

## Results

Cases for immunohistochemical technique include 40 males (66.7%) and 20 females (33.3%). The mean age for males was of 60.95±7.79 years and the mean age for females was of 57.35±8 years. No statistically significant differences were detected between their mean ages (p > 0.05). 

Cases for *IL6 C/G* gene polymorphism: HCV cases include 26 (46.7%) males and 29 (53.3%) females; age ranged from 34-67 years and the mean age was of 44.3 ±13 years. HCC cases include 42 (70%) males and 18 (30%) females; age ranged from 48-60 years and the mean age was of 46.8±15.9 years. Controls include 40 (80%) males and 10 (20%) females; age ranged from 32-57 years and the mean age was of 46.7±13.3 years.

**Figure 1:**
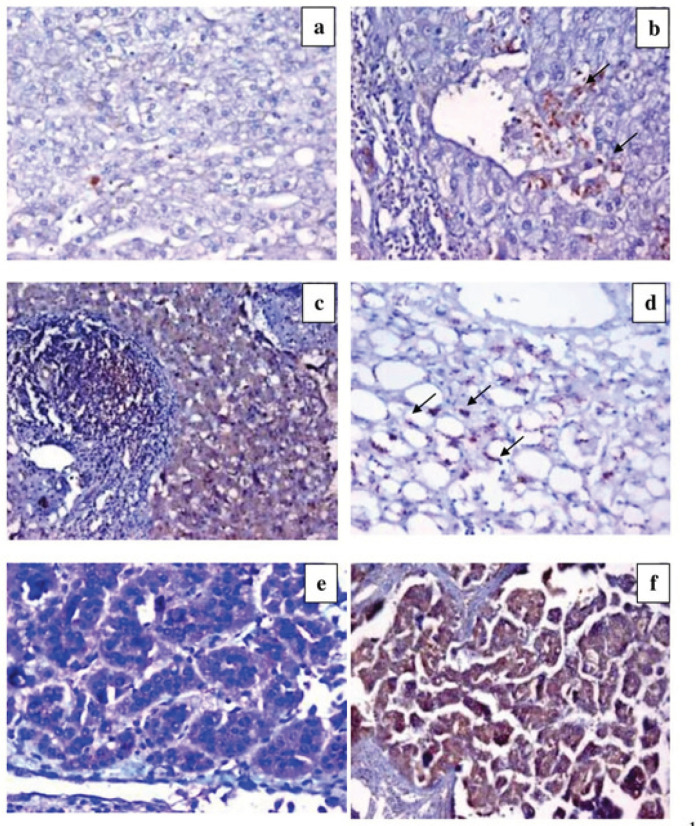
IL-4 Expression. a) Section in chronic HCV with low grade activity and low score of fibrosis, shows negative expression of IL-4 (DAB ChromogenX200). b) Section in chronic HCV with moderate activity and moderate score of fibrosis shows focal positive IL-4 expression in macrophages (DAB ChromogenX200). c) Section in chronic HCV with moderate grade activity and moderate score of fibrosis, shows diffuse mild hepatocytic IL-4 expression (DAB ChromogenX200). d) Section in chronic HCV with marked steatosis shows marked positive IL-4 expression (arrows) (DAB ChromogenX200). e) Section in low grade HCC, showing diffuse mild hepatocytic IL4 expression (DAB chromogenX400). f) Section in high grade HCC, shows diffuse strong hepatocytic IL-4 expression (DAB chromogenX400).

**Figure 2:**
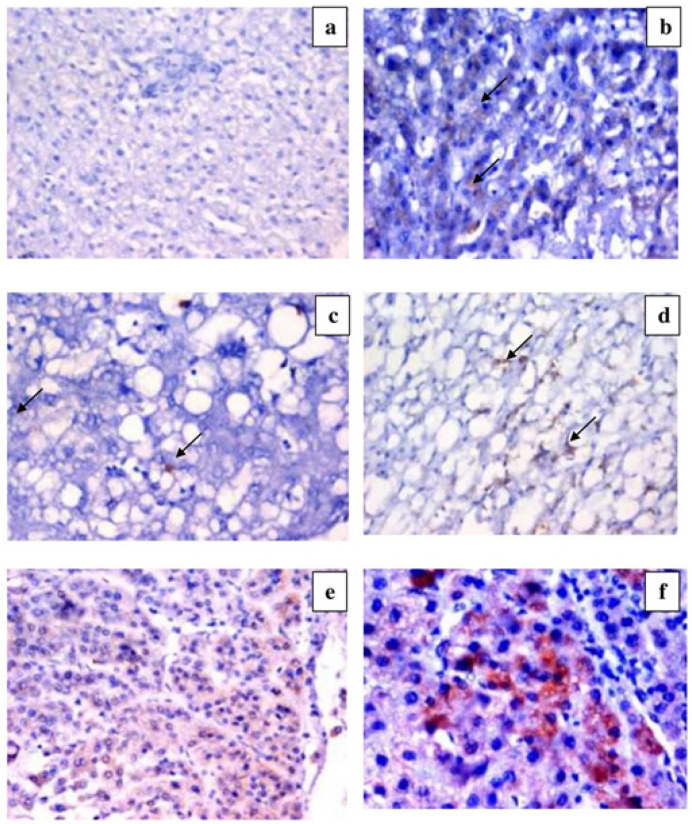
IL-17 Expression. h low grade activity and low score of fibrosis, shows negative expression of IL-17 (DAB ChromogenX200). b) Section in chronic HCV with moderate activity shows focal positive IL-17 expression in hepatocytes and macrophagAes (arrows) (DAB ChromogenX200). c) Section in chronic HCV with mild degree of steatosis, shows low expression of IL-17 (arrows) (DAB ChromogenX200). d) Section in chronic HCV with marked steatosis shows positive expression of IL-17 in macrophages (arrows) (DAB ChromogenX200). e) Section in low grade HCC shows diffuse mild hepatocytic IL-17 expression (DAB chromogenX400). f) Section in high grade HCC shows strong hepatocytic IL-17 expression (DAB chromogeX400).

**Figure 3.**
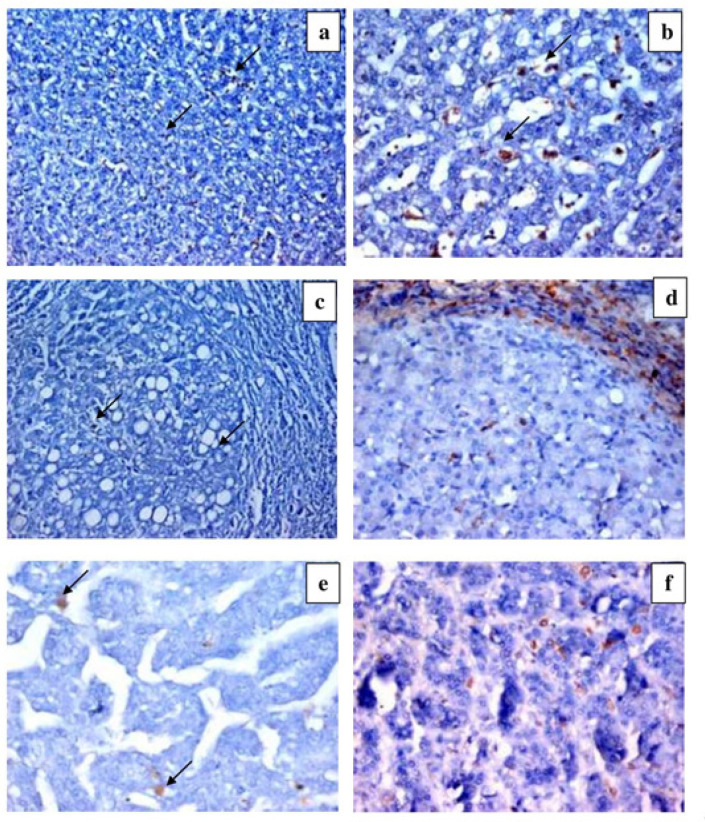
CD163 Expression. a) Section in chronic HCV with low grade activity and low score of fibrosis, shows positive CD163 expression in scattered macrophages (DAB ChromogenX100). b) Section in chronic HCV with high hepatitis activity and low score of fibrosis, shows positive CD 163 expression in scattered macrophages (DAB ChromogenX200). c) Section in chronic HCV with cirrhosis shows scanty positive CD163 expression in few macrophages (arrows) (DAB ChromogenX200). d) Section in chronic HCV with moderate hepatitis activity and cirrhotic pattern shows positive CD163 expression in scattered macrophages within the hepatic lobule and fibrous septa (DAB ChromogenX200). e) Section in low grade HCC shows positive CD163 expression in few scattered macrophages (arrows) (DAB ChromogenX200). f) Section in high grade HCC shows positive CD163 expression in many scattered macrophages (DAB ChromogenX200).

The level of IL-4, IL-17 and CD163 was positively correlated with the grade of hepatitis activity; marked grades of hepatitis activity showed almost the highest degree of expression, and vice versa (Table 1) (Figures 1-3).

As regards hepatic fibrosis, our results showed that cases of post-hepatitis fibrosis of high scores (F3 and F4) showed higher degrees of *IL-4, IL-17*, and *CD163 *expression, compared to low scores of hepatic fibrosis (F1 and F2) (Table 2) (Figures 1-3).

Our results showed a positive relation between the degree of hepatic steatosis and the expression profile of *IL-4, IL-47*, and *CD163* (Table 3) (Figures 1-3).

High grade HCC showed higher percentage of hepatocytes which express IL-4 and CD163 in their cytoplasm than low grade HCC; however, *IL-17 *expression was higher in low grade HCC (Table 4) (Figures 1-3).

*IL6 C/G* gene polymorphism results showed that there was a difference in allele frequencies between patients with HCV and control group (p=0.1). Moreover, there was a difference between allele frequencies between HCC and control group (p=0.3), though these differences were insignificant. G allele was remarkably “yet insignificantly” frequent among HCV and HCC patients (86.4% and 84.2% respectively) compared to the controls (76.0%), on the other hand, C allele frequency was similar amid all the studied groups. In addition, the G/G genotype was more frequent among HCV patients (78.2%) and HCC patients (75%) in comparison with the controls (62%) while the frequency of C/C genotype was low among HCV patients (5.5%), HCC patients (6.7%) and controls (10.0%) (Table 5).

**Table 1:** Expression Profile of IL-4, IL-17 and CD163 in Different Grades of Hepatitis Activity in Liver Sections of Chronic Hepatitis C

		IL4 Expression			IL-17 Expression			CD163 Expression			Total n.
		Negative n. (%)	Low n. (%)	High n. (%)	Negative n. (%)	High n. (%)	High n. (%)	Negative n. (%)	Low n. (%)	High n. (%)	
Hepatitis activity	mild	8 (53.33)	4 (26.67)	3 (20.0)	5a (33.3)	5b (33.3)	5b (33.3)	8a (53.3)	3a (20.0)	4a (26.7)	15
	moderate	5 (16.67)	10 (33.33)	15 (50.0)	0a (0.0)	10b (33.3)	20a, b (66.7)	13a (43.3)	9a (30.0)	8a (26.7)	30
	marked	1 (6.67)	1 (6.67)	13 (86.66)	0a, b (0.0)	0b (0.0)	15 (100)	1a (6.7)	2a, b (13.3)	12b (80.0)	15
Total		14 (23.33)	15 (25.0)	31(51.67)	5 (8.3)	15 (25.0)	40 (66.7)	22 (36.7)	14 (23.3)	24 (40.0)	60

Each subscript letter denotes a subset of IL-17 intensity categories whose column proportions do not differ significantly from each other at the 0.05 level.

**Table 2:** Expression Profile of IL-4, IL-17 and CD163 in Different Stages of Fibrosis in Liver Sections of Chronic Hepatitis C

		IL4 Expression			IL-17 Expression			CD163 Expression			Total n.
		Negative n. (%)	Low n. (%)	High n. (%)	Negative n. (%)	High n. (%)	High n. (%)	Negative n. (%)	Low n. (%)	High n. (%)	
Hepatic Fibrosis	Mild/Moderate	10a (33.33)	9b (30.0)	11b (36.67)	5a (16.7)	10a, b (33.3)	15b (50.0)	19a (63.33)	6a, b (20.0)	5b (16.67)	30
	Marked/Cirrhosis	4a (13.33)	6b (20.0)	20a, b (66.67)	0a (0.0)	5a, b (16.7)	25b (83.3)	3a (10.0)	8a, b (26.67)	19b (63.33)	30
Total		14 (23.33)	15 (25.0)	31 (51.67)	5 (8.3)	15 (25.0)	40 (66.7)	22 (36.7)	14 (23.3)	24 (40.0)	60

**Table 3:** Expression Profile of IL-4, IL-17 and CD163 in Different Grades of Hepatic Steatosis in Liver Sections of Chronic Hepatitis C

		*IL4 *Expression			*IL-17* Expression			*CD163* Expression			Total (n.)
		Negative n. (%)	Low n. (%)	High n. (%)	Negative n. (%)	High n. (%)	High n. (%)	Negative n. (%)	Low n. (%)	High n. (%)	
Hepatic Steatosis	mild	8a 66.66)	2b (16.67)	2b (16.67)	3a (25)	7b (58.33)	2b (16.67)	6a (50)	2a (16.67)	4a (33.33)	12
	moderate	5a (16.67)	10b (33.33)	15a, b(50)	2a(6.67)	8b (26.66)	20a, b (60.67)	9a (30)	8a (26.67)	13a (43.33)	30
	marked	1a, b (5.55)	3b(16.67)	14 (77.78)	0a, b(0)	0b (0)	18a (100)	7a (38.88)	4a, b (22.22)	7b (38.88)	18
Total		14 (23.33)	15 (25)	31 (51.67)	5(8.3)	15 (25)	40 (66.7)	22 (36.7)	14 (23.3)	24 (40)	60

Each subscript letter denotes a subset of IL17 Intensity categories whose column proportions do not differ significantly from each other at the 0.05 level

**Table 4:** Expression Profile of IL-4, IL-17 and CD163 in Studied Grades of HCC

		*IL4* Expression			*IL-17* Expression			*CD163* Expression			Total (n.)
		Negative n. (%)	Low n. (%)	High n. (%)	Negative n. (%)	High n. (%)	High n. (%)	Negative n. (%)	Low n. (%)	High n. (%)	
HCC grade	low grade	10a, b (25)	21b (52.5)	9a (22.5)	5 (12.5)	15 (37.5)	20 (50)	29a (72.5)	11a (27.5)	0b	40
	High grade	4a, b (20)	2b (10)	14a (70)	7 (35)	9 (45)	4 (20)	10a (50)	2a (10)	8b (40)	20
Total HCC		14 (23.3)	23 (38.3)	23 (38.3)	12 (20)	24 (40)	24 (40)	39 (65)	13 (21.7)	8 (13.3)	60

Each subscript letter denotes a subset of IL17 Intensity categories whose column proportions do not differ significantly from each other at the 0.05 level

**Table 5:** Frequency of IL6 Allele and Genotype among All Studied Groups

		Groups						
		Control N=50	HCV N=55	HCC N=60	Total	p value		
		n. (%)	n. (%)	n. (%)		Control vs HCV	Control vs HCC	HCV Vs HCC
IL6 polymorphism	Hetero G/C	14 (28)	9 (16.4)	11 (18.3)	34	0.1	0.3	0.9
	Homo G/G	31 (62)	43 (78.2)	45 (75)	119			
	Homo C/C	5 (10)	3 (5.5)	4 (6.7)	12			
allele	G allele	76 (76)	95 (86.4)	101 (84.2)	272			
	C allele	24 (24)	15 (13.6)	19 (15.8)	58			

## Discussion

HCC represents a classic paradigm of inflammation-linked cancer. Immediately following a viral infection, a strong host response -represented by cytokine production - is initiated (Dondeti et al., 2016). 

In our study, we found high expression of* IL-4* in higher grades of hepatitis activity and late stages of fibrosis. This is parallel with the findings of Mourtzikou et al., (2014) where *IL-4 *expression correlates with the degree of necro-inflammation and advanced stages of fibrosis with maximal IL-4 levels were observed in hepatic cirrhosis. Also, Batsaikhan et al., (2019) concluded that IL-4 is an important cytokine in predicting advanced liver fibrosis in HCV-infected patients. In addition, there is an increased expression of *IL-4* in higher degrees of hepatic steatosis. This can be attributed to activation of Kupffer cells, which is evidenced in chronic viral hepatitis, then, these activated cells secrete proinflammatory mediators which in turn initiate fatty liver disease and steatosis (Gao et al., 2009). 

Regarding our studied HCC specimens, we found a significant high *IL-4* expression in higher grades of HCC compared to lower grades. According to Wu et al., (2015), there is suggested evidence of an association between* IL-4* expression with HCC as well as HCV. IL-4 can promote development of specific immune cell subtypes, such as macrophages, B cells, CD4+Th2 cells, and CD8+ T cells, which play a pivotal role in the tumor progression of inflammation-related cancers, including HCC (Capece et al., 2013). Furthermore, Kogame et al., (2016) stated that IL-4 in the tumor microenvironment plays an important role in recurrence of HCC.

Our study demonstrated a positive correlation between *IL-17* expression with marked grades of hepatitis activity and high scores of liver fibrosis compared to lower ones. Tan et al., (2013) reported that IL-17has critical role in the pathogenesis of liver fibrosis, as *IL-17* promoted proinflammatory cytokine expression, neutrophil influx, liver injury, inflammation, and fibrosis through hepatic stellate cell activation, so, there is increased level of IL-17 with increasing inflammation, fibrosis and cirrhosis. Woltman et al., (2000) reported that intrahepatic* IL-17 *expression was positively correlated with serum indices of hepatic fibrosis. Furthermore, other studies detected a significant increase in serum IL-17 levels in HCV-patients with or without cirrhosis and found significant higher values in cirrhotic states that correlated well with the severity of the cirrhosis (Jimenez-Sousa et al., 2010; Zhao et al., 2011; Fathy et al., 2011). We found a positive relation between expression of *IL-17* and the degree of hepatic steatosis. This agrees with Gomes et al., (2016) who suggested that, IL-17 may initiate steatohepatitis progressing to HCC. 

In accordance with Gomes et al., (2016) who stated that IL-17 was present in an early stage of HCC, we found more predominant expression of *IL-17* in low grade HCCs. This may indicate that the immune function was suppressed in HCC patients. Hammad et al., (2013) reported a significant progressive elevation in circulating IL-17 levels that were concomitant with the progression of hepatic exacerbation from HCV-induced liver fibrosis to cirrhosis and finally to HCC. Furthermore, Zhang et al., (2009) demonstrated that IL-17+ T cells were discovered in large numbers within HCC and were associated with poor survival and recurrence, suggesting that IL-17 could facilitate HCC progression. However, the direct effect and potential mechanism of IL-17 in regulating human HCC cell growth remain incompletely defined.

Current study revealed increased expression of *CD163 *in high inflammatory grades, high fibrotic stages, and marked degree of steatosis of chronic HCV. Several studies have demonstrated that M2 macrophages, with CD163 is one of the M2 lineage, secrete immunosuppressive cytokines, inhibitory molecules and lack co-stimulatory molecules, thus promoting T helper (Th)-1 impairment and viral persistence in chronic viral infections. Additionally, M2 macrophages secrete pro-fibrotic cytokines and factors, thus promoting tissue fibrosis and neoplasia (Murray and Wynn, 2011). In support to our findings, Kazankov et al., (2014) found higher levels of sCD163 in patients with advanced fibrosis compared to patients with mild fibrosis, moreover, the patients who had milder disease still had elevated sCD163, suggesting macrophage activation even in moderate disease, albeit to a lower extent. Another study by Dolganiuc et al., (2007) showed increased CD163 mRNA levels in the livers of patients with chronic HCV infection. Also, Hiraoka et al., (2005) found increased surface-bound *CD163* expression and soluble *CD163* plasma levels in acute and chronic viral hepatitis. We also detected a positive relation between the degree of hepatic steatosis and *CD163* expression. The same relation was reported by Mueller et al., (2015).

We found higher expression of *CD136* in macrophages of advanced grades of HCC compared with lower grades. Raggi et al., (2017) reported that macrophages of HCC carry large number of CD163 and Kazankov et al., (2016) observed increased plasma concentration of sCD163 in HCC and its association with worse outcome. On the contrary, Kong et al., (2013) found few *CD163*- expressing cells in HCC. Although studies reported poor prognosis in patients with *CD163*- expressing cells in breast cancer (Shabo et al., 2008) and rectal cancer (Shabo et al., 2009); another study reported favorable clinicopathological features in colon cancer (Koelzer et al., 2015). Controversies still exist regarding role of CD163 in cancer progression.

Regarding* IL6-572 C/G* gene polymorphism, we found no significant difference in the frequency of G/C, G/G, and C/C between HCV, HCC patients, and the controls. There was a higher frequency of G allele among HCV and HCC patients in comparison to controls, yet this finding was insignificant. These findings can conclude that IL6 -572 G/C polymorphism could not be intensely associated with HCC susceptibility. 

Our results were concomitant with a study made by Falleti et al., (2009) which found no association between *IL6-572* gene polymorphism and occurrence of HCC. Moreover, Cussigh et al., (2011) has confirmed that there is no link between* IL6-572* promoter gene polymorphism and HCC occurrence. Furthermore, a meta-analysis has been executed by Liu et al., (2014) on *IL6* gene polymorphism and risk of HCC development concluded that IL-6-174 G/C, but not −572 G/C polymorphism could lead to HCC predisposition. On the other hand, a study by Sghaier et al., (2017) validated that IL-6-572 G/G genotype was more frequent in HCV patients compared to HCC patients, which was contradicting our results.

The main limitation of our study is the relatively small number of studied cases, so number of cases in each group in terms of hepatitis activity and fibrosis was limited.

In conclusion, our study demonstrated association between hepatic expression of *IL-4, IL-17*, and* CD163* with severity of HCV, being more expressed in moderate and marked grades of activity, late stages of fibrosis, and higher degrees of steatosis. Meanwhile, milder disease also showed expression but to a lower extent. This finding suggests the important role of these cytokines in the development of HCC so blocking of these proteins might be promising targeted therapy in chronic HCV to control progression to cirrhosis then to HCC. On the other hand,* IL6-572* promoter gene polymorphism might not be a useful determinant in predicting the outcome of HCV infection or HCC susceptibility. 

## Author Contribution Statement

TA conceived, designed, and revised the manuscript. ME, GR reviewed all tissue samples and drafted the manuscript. KR Acquisition of data and providing clinical data of patients. OH handled and interpreted the patient data. RF reviewed blood samples and drafted the manuscript, NSH interpreted all data and wrote the manuscript. All authors read and approved the final manuscript.
